# Enhanced digital pathology image recognition via multi-attention mechanisms: the MACC-Net approach

**DOI:** 10.1038/s41598-025-17369-4

**Published:** 2025-08-25

**Authors:** Feng Liu, Zheng Wang, Baotian Li, Decai Wang, Mingyu Liu, Fangfang Gou, Jia Wu

**Affiliations:** 1 School of Information Engineering, Shandong Youth University of Political Science, Jinan, China; 2 New Technology Research and Development Center of Intelligent Information Controlling in Universities of Shandong, Jinan, China; 3 Smart Healthcare Big Data Engineering and Ubiquitous Computing Characteristic Laboratory in Universities of Shandong, Jinan, China; 4 Weifang Engineering Vocational College, Qingzhou, China; 5 State Key Laboratory of Public Big Data, College of Computer Science and Technology, Guizhou University, Guiyang, 550025 China; 6 Research Center for Artificial Intelligence, Monash University, Clayton, Melbourne, VIC 3800 Australia; 7 Sdu-Anu Joint Science College, Shandong University, Weihai, 264209 China

**Keywords:** Multiple attention, Digital pathology images, Multi-scale aggregation, Image segmentation, Auxiliary diagnosis, Cancer, Computational biology and bioinformatics, Engineering, Mathematics and computing

## Abstract

Digital pathology has revolutionized cancer diagnosis through microscopic analysis, yet manual interpretation remains hindered by inefficiency and subjectivity. Existing deep models for osteosarcoma cell nucleus recognition suffer from the difficulty of capturing hierarchical relationships in single-dimensional attention mechanisms, leading to inaccurate edge recognition. Furthermore, the fixed receptive field of CNNs limits the aggregation of multi-scale information, hindering the differentiation of overlapping cells. This study introduces MACC-Net, a novel multi-attention based method designed to enhance the recognition accuracy of digital pathology images. By integrating channel, spatial, and pixel-level attention mechanisms, MACC-Net overcomes the limitations of traditional single-dimensional attention models, improving feature consistency and receptive field expansion. Experimental results demonstrate a Dice Similarity Coefficient (DSC) of 0.847, highlighting MACC-Net’s potential as a reliable auxiliary diagnostic tool for pathologists. Code: https://github.com/GFF1228/MACCNet.

## Introduction

Pathological diagnosis is the “gold standard” for tumor diagnosis, and its accuracy directly impacts clinical treatment decisions^1^. Pathologists use microscopes to observe cell morphology and distribution, distinguishing between normal cells, inflammatory cells, and cancer cells, and determine the presence, type, grade, and depth of invasion of tumors^2,3^. This is particularly true for the diagnosis of highly malignant bone tumors such as osteosarcoma, where accurate pathological analysis directly influences surgical and chemotherapy planning. Therefore, this study used digital pathology images of osteosarcoma as a test target to validate the model’s performance.

Whole-slide imaging (WSI) technology has enabled the digitization of pathology images, but manual interpretation still faces challenges such as low efficiency, high subjectivity, and decreased diagnostic consistency due to fatigue^4–6^. For complex pathology images, pathologists must switch between different magnifications to identify key areas and make judgments, a time-consuming and error-prone process^7,8^. Pathology diagnosis is expensive in terms of equipment, labor, and time, making these challenges even more pronounced in resource-limited settings^9^.

Artificial intelligence technology provides a new approach to improve this situation^10–12^. Through image segmentation algorithms, artificial intelligence systems can decompose complex pathological images into different tissue components, such as tumor tissue and normal tissue, thereby improving diagnostic efficiency^13,14^. Among them, convolutional neural networks (CNNs) have achieved remarkable success in tasks such as cell nucleus segmentation, tumor grading, and metastasis detection^15–17^. Attention mechanisms perform well in enhancing the interpretability of models by focusing on diagnostically relevant areas^18–20^. For example, Li Xiaorong et al.^17^ proposed a CNN architecture that combines iterative attention feature fusion and residual modules, aiming to accurately segment overlapping and multi-scale cell nuclei.

However, existing deep learning models still have many limitations in the recognition of osteosarcoma pathology images^21^. The single-dimensional attention mechanism cannot fully identify the hierarchical relationship between osteosarcoma cell nuclear features and the global pathological tissue background, resulting in low accuracy in cell nuclear edge recognition^22,23^. Osteosarcoma pathology images contain many overlapping cell regions. The fixed receptive field of CNNs hinders the aggregation of multi-scale background information, making it difficult to distinguish overlapping and aggregated cells and tissues^24^.

Based on this, this study is based on the Multiple Attention Guided Assisted Recognition of Digital Pathology Images (MACC-Net), which aims to effectively assist doctors to accurately recognize cancerous tissues in digital pathology images. Its core innovation mainly consists of three modules: hybrid attention feature extraction module (HAFEM), cascade context fusion module (CCIFM), and attention map dynamic balancing module (AMDBM). The HAFEM module maintains nuclear feature consistency through fused channel, spatial, and pixel-point attentions in histopathological imagery. The CCIFM preserves feature uniformity by employing adaptive average pooling and non-local operations to capture global context and expand the model’s receptive field for multi-scale integration. Meanwhile, the AMDBM dynamically modulates feature weights via fused foreground, background, and boundary attention maps, prioritizing target-relevant features while suppressing irrelevant information. MACC-Net overcomes the representation bottleneck of single-dimensional attention and expands the model’s receptive field, enabling the fusion of multi-scale contextual information and significantly improving the accuracy of cell nucleus edge recognition. It can more accurately identify pathological images of cancerous tissue, providing doctors with a reliable auxiliary diagnostic tool.

The rest of this paper is organized as follows: “Methods” introduces the proposed method in detail, “Results” presents the experimental results, “Discussion” discusses and analyzes the proposed method in relation to current research, and “Conclusion and outlook” summarizes the clinical implications and future directions.

## Methods

**Fig. 1:**
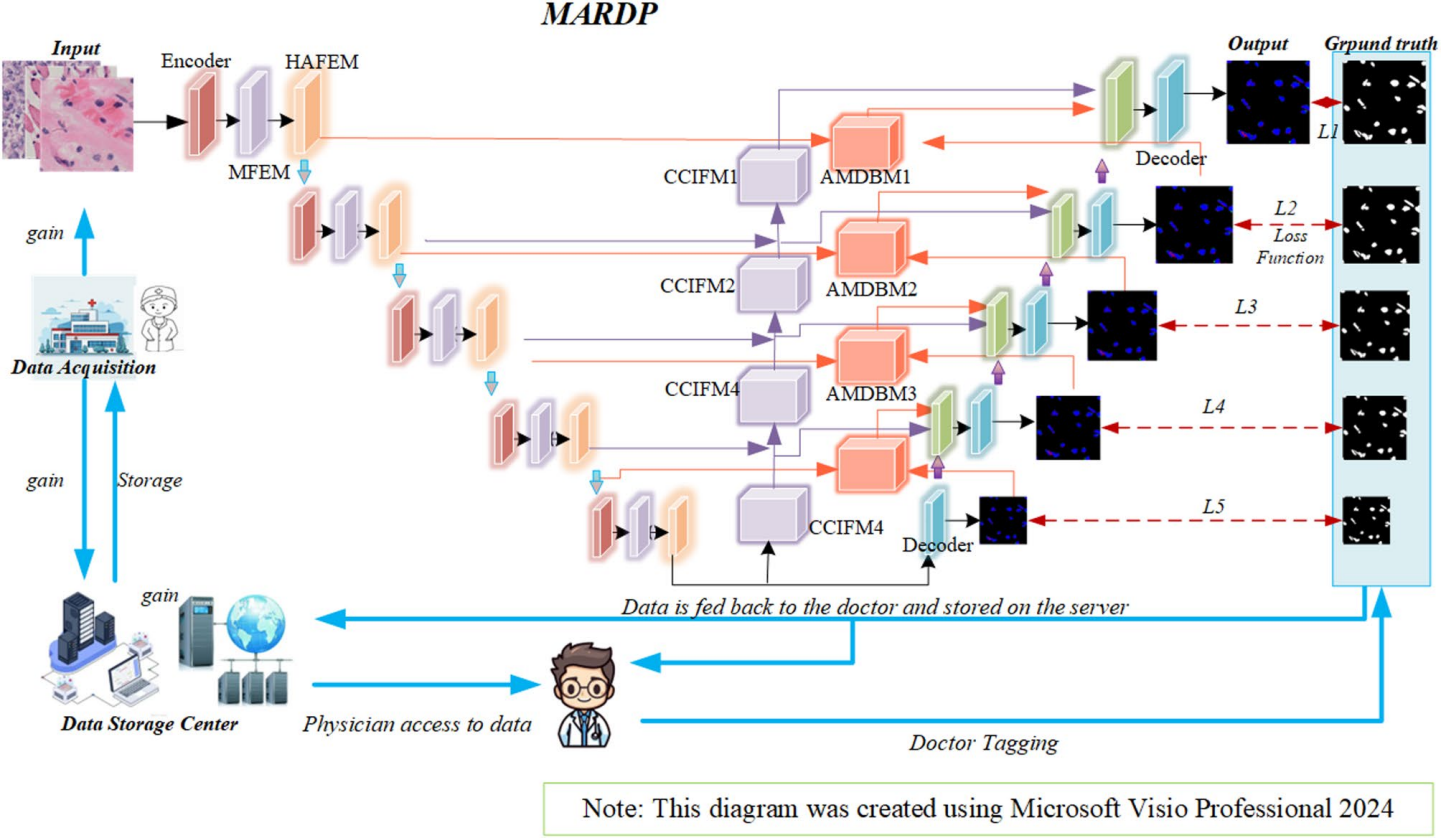
Overall architecture of MACC-Net.

This study proposes a multi-head attention mechanism-based digital pathology image assisted recognition network (MACC-Net) to achieve accurate segmentation of cell nuclei in pathological sections. The overall network structure is shown in Fig. 1. MACC-Net mainly consists of the following four modules.

1. The Multi-scale Feature Extraction Module (MFEM) extracts semantic features at different scales using ResNet, capturing tissue representations across various hierarchical levels.
2. The Hybrid Attention Feature Enhancement Module (HAFEM) simultaneously enhances lesion features using channel and spatial attention mechanisms, suppressing background interference.
3. The Cascaded Context Integration and Fusion Module (CCIFM) performs semantic cascading of features at each scale, expands the receptive field via dilated convolutions, and enables large-scale contextual modeling.
4. The Attention-Modulated Dynamic Balancing Module (AMDBM) dynamically adjusts feature weights by calculating and fusing foreground, background, and boundary attention maps. This enhances focus on task-relevant features while suppressing irrelevant information.

### Multi-scale feature extraction module (MFEM)

In digital pathological images, cells exhibit significant morphological heterogeneity, manifested not only as structural variations across multiple scales but also as prominent characteristics (e.g., blurred nuclear edges and discontinuous textures). These complexities substantially increase the difficulty of segmentation tasks^25–27^. To address this challenge, effectively extracting and fusing multi-scale feature information has become a critical research focus. In this paper, we propose a Multi-scale Feature Extraction Module (MFEM) to enhance the model’s ability to perceive tumor regions at diverse scales.

The MFEM module is based on a lightweight backbone network and adopts a hierarchical feature extraction approach to capture multi-scale information from low-level textures to high-level semantics layer by layer. Assuming the input pathological image is $\:I\in\:{\mathbb{R}}^{H\times\:W\times\:3}$, the backbone network is first used to extract four-level semantic feature representations:

1 $$\:\{{F}_{1},{F}_{2},{F}_{3},{F}_{4}\}=Backbone\left(I\right)$$

Among them, $\:{F}_{i}\in\:{\mathbb{R}}^{{H}_{i}\times\:{W}_{i}\times\:{C}_{i}}$ respectively represent the feature maps extracted at different scales. As the network hierarchy deepens, the size of the feature maps gradually decreases while the semantics are increasingly enhanced. Specifically, $\:{F}_{1}$ captures the edge and texture features in the original image, $\:{F}_{2}$ and $\:{F}_{3}$ focus on the local structures and mid-level semantics, and $\:{F}_{4}$ represents the global high-level semantic abstraction.

The MFEM enhances multi-scale perception by alternating dilated and strided convolutions, balancing receptive field size and computational efficiency. This adaptability is crucial for lesions with varying morphologies and densities. Additionally, shallow features are retained to improve nuclear boundary detection, particularly for small or irregular nuclei.

Additionally, to avoid excessive loss of information flow during the downsampling process, the MFEM module draws inspiration from the U-Net architecture design concept and retains a skip connection mechanism during feature extraction. This enables shallower-layer features to re-participate in the feature fusion process during subsequent fusion stages, effectively preserving edge and texture information of tumor regions.

Batch Normalization and ReLU activation functions are introduced after the extraction of each level of feature maps, ensuring stable gradient propagation and nonlinear enhancement during the training process. The specific feature extraction process for each level can be expressed as:

2 $$\:{F}_{i}=ReLU\left(BN\left({Conv}_{3\times\:3}\left({F}_{i-1}\right)\right)\right)$$

Among them, $\:{Conv}_{3\times\:3}$ denotes standard convolution operations, BN represents batch normalization operations, and ReLU refers to activation functions.

### Multi-attention enhancement module (HAFEM)

**Fig. 2:**
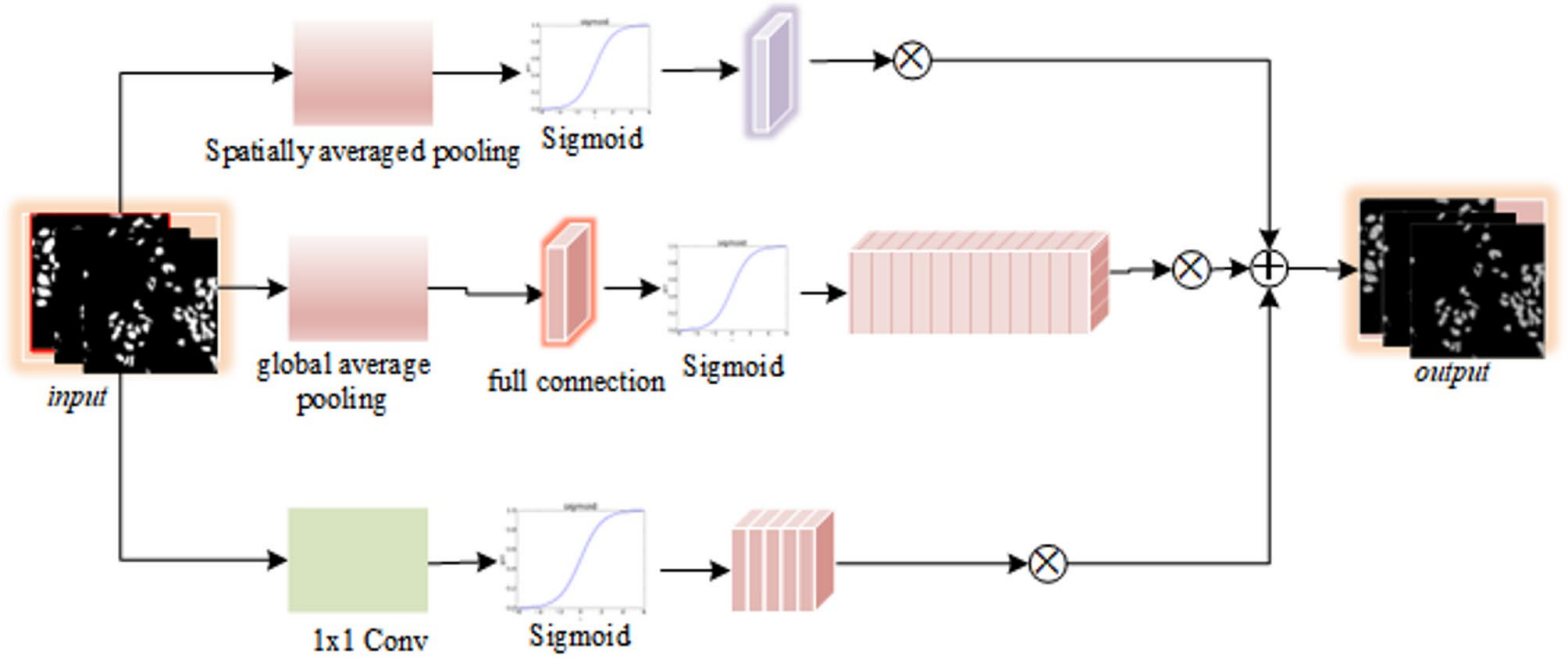
HAFEM module structure diagram.

Digital pathology images contain a rich background of non-lesional tissues^28–30^. CNN’s receptive field and local feature extraction capabilities are insufficient, making it difficult to effectively distinguish tumor areas. To this end, this paper introduces a multi-attention enhancement module (HAFEM) to enhance the network’s response to salient cell tissue regions and improve the expression selectivity of feature channels. As shown in Fig. 2, The HAFEM module is primarily composed of three complementary attention mechanisms: Channel Attention focuses on “which feature channels are important,” while Spatial Attention emphasizes “which positions in the image are more critical,” extracting nuclear boundary features. Pixel-wise Attention captures features of smaller nuclei or inter-nuclear gaps. This module applies weighted calculations of channel and spatial dimensions to each input feature map $\:{F}_{i}\in\:{\mathbb{R}}^{{H}_{i}\times\:{W}_{i}\times\:{C}_{i}}$, resulting in an enhanced representation $\:{F}_{i}^{{\prime\:}}$.

3 $$\:F_{i}^{{\prime \:}} = {\text{Con}}_{{1 \times \:1}} \left( {F_{i}^{{\left( s \right)}} \oplus F_{i}^{{\left( c \right)}} \oplus F_{i}^{{\left( p \right)}} } \right),{\text{i}} = {\text{1,2}},{\text{3,4}},5$$

Among them, $\:{F}_{i}^{{\prime\:}}$ denotes the unified attention feature map generated by the multi-attention module, while $\:{\text{C}\text{o}\text{n}}_{1\times\:1}$ reduces feature channel dimensionality following the activation layer, $\:{F}_{i}^{\left(s\right)}$ is the spatial attention feature of the pathological image, $\:{F}_{i}^{\left(c\right)}$ is the channel attention feature of the pathological image, and $\:{F}_{i}^{\left(p\right)}$ is the pixel-wise attention feature of the pathological image.

The channel attention module leverages global contextual information to guide the importance modeling of feature channels. Specifically, global average pooling and max pooling are first performed on each channel to form two one-dimensional descriptive vectors.

4 $$\:{f}_{avg}=\text{A}\text{v}\text{g}\text{P}\text{o}\text{o}\text{l}\left({F}_{i}\right)$$

5 $$\:{f}_{max}=\text{M}\text{a}\text{x}\text{P}\text{o}\text{o}\text{l}\left({F}_{i}\right)$$

Subsequently, a shared multi-layer perceptron (MLP) is used for non-linear mapping, outputting the channel attention weight vector $\:{M}_{c}$.

6 $$\:{M}_{c}=\sigma\:\left(\text{M}\text{L}\text{P}\left({f}_{avg}\right)+\text{M}\text{L}\text{P}\left({f}_{max}\right)\right)$$

The final channel attention response is expressed as:

7 $$\:{F}_{i}^{\left(c\right)}={M}_{c}\otimes\:{F}_{i}$$

where $\:{\upsigma\:}$ denotes the Sigmoid activation function, and $\:\otimes\:$ represents the element-wise multiplication operation between channels.

To further enhance the discriminability of features in spatial positions, the HAFEM module introduces a Spatial Attention mechanism, which aims to detect the most discriminative regional positions in the image. Channel-attentive feature maps undergo max pooling and average pooling operations along the channel axis, producing two distinct spatial context maps.

8 $$\:{f}_{avg}^{s}={\text{A}\text{v}\text{g}\text{P}\text{o}\text{o}\text{l}}_{c}\left({F}_{i}^{\left(c\right)}\right)$$

9 $$\:{f}_{max}^{s}={\text{M}\text{a}\text{x}\text{P}\text{o}\text{o}\text{l}}_{c}\left({F}_{i}^{\left(c\right)}\right)$$

Both maps undergo concatenation before being processed by a 7 × 7 convolutional layer, yielding the spatial attention map $\:{M}_{s}$.

10 $$\:{M}_{s}=\sigma\:\left({\text{C}\text{o}\text{n}\text{v}}_{7\times\:7}\left(\left[{f}_{avg}^{s};{f}_{max}^{s}\right]\right)\right)$$

The final spatially enhanced feature representation $\:{F}_{i}^{\left(s\right)}$ is expressed as:

11 $$\:{F}_{i}^{\left(s\right)}={M}_{s}\otimes\:{F}_{i}^{\left(c\right)}$$

The pixel-wise attention feature $\:{F}_{i}^{\left(p\right)}$ is calculated as:

12 $$\:{F}_{i}^{\left(p\right)}=\sigma\:\left({Conv}_{1\times\:1}\left({e}_{i}\right)\right),i=\text{1,2},\text{3,4},5$$

The three attention mechanisms described above respectively model the semantic importance of channel dimensions, the saliency of spatial regions, and the significance of small-scale tissue features, thereby collaboratively enhancing the model’s focusing ability on cell nucleus regions and suppressing background interference. In terms of structure, the HAFEM module is lightweight with a small number of parameters, making it easy to couple with the backbone network and suitable for promotion in high-resolution pathological image scenarios.

### Cascaded context integration and fusion module (CCIFM)

**Fig. 3:**
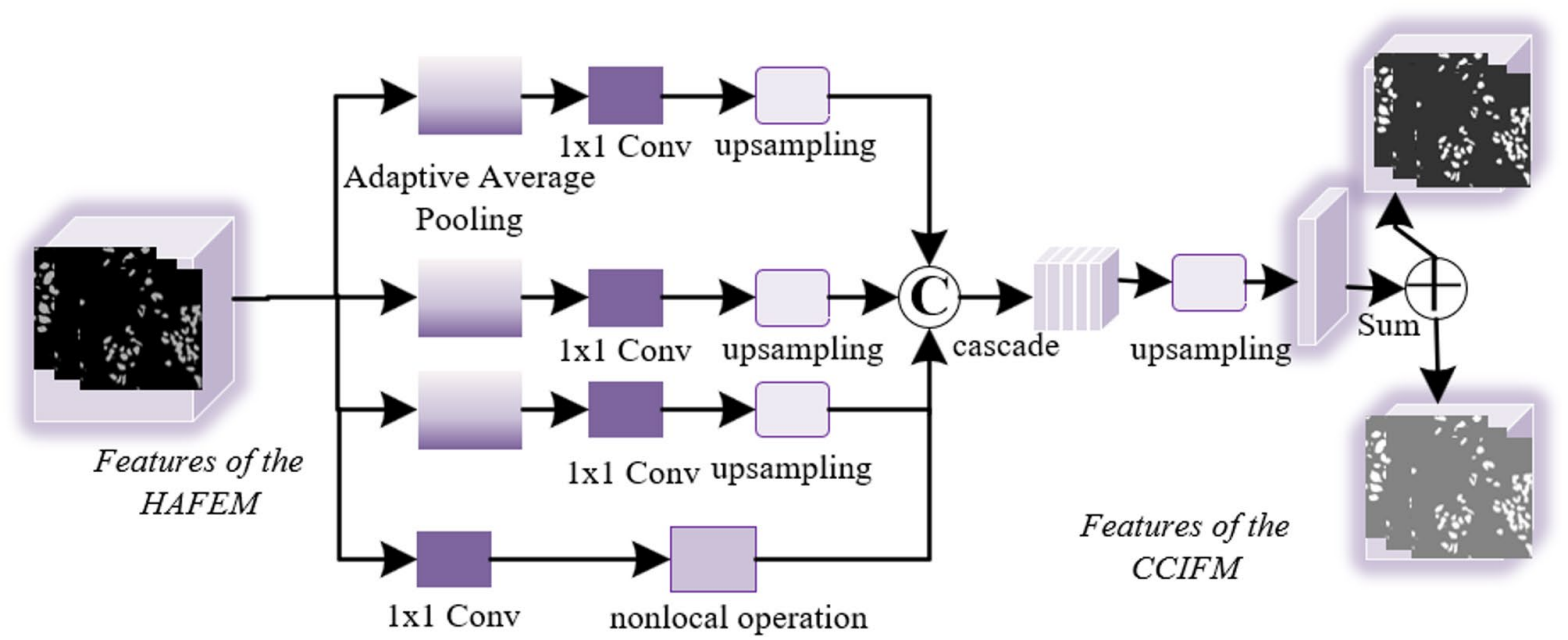
Structure of the CCIFM module.

Cell nuclei exhibit diverse morphologies, irregular distribution, blurred boundaries, and clustered or overlapping distributions, making their differentiation heavily dependent on contextual information^31,32^. After multi-scale feature extraction and attention enhancement, it becomes crucial to further integrate information from different levels to build a globally consistent understanding of the lesion. To this end, this paper designs a Cascaded Context Integration and Fusion Module (CCIFM) to effectively fuse shallow and deep semantics, enhance the modeling of contextual dependencies, and thus improve the structural integrity and boundary accuracy of the final segmentation prediction. The detailed structure of the CCIFM module is shown in Fig. 3, which feeds the multi-scale feature maps $\:{F}_{i}^{{\prime\:}}$ from the HAFEM module into four branches for operations to extract global contextual information. The initial three branches first perform adaptive average pooling on feature map $\:{F}_{i+1}^{{\prime\:}}$​, with pooling kernel dimensions defined as:

13 $$\:{F}_{c}AdPdo\left(L,j\right)=(2j-1)(6-L)$$

14 $$\:L=\text{1,2},\text{3,4},j=\text{1,2},3$$

Here, *L* indicates the total layers in the CCIFM module, while *j* corresponds to the index of its first three branches. Each initial branch employs 1 × 1 convolutions to compress feature channels to 25% of original dimensionality, followed by upsampling to restore spatial resolution. For branch 4, a 1 × 1 convolution embeds each feature map pixel into vector space, where non-local operations model pairwise pixel relationships to capture long-range dependencies, ultimately deriving deep semantic feature representations.

Finally, assuming the upsampling operation is $\:\mathcal{U}\left(\bullet\:\right)$, the fused features can be expressed as:

15 $$\:{F}_{c}=\text{C}\text{o}\text{n}\text{c}\text{a}\text{t}\left(\mathcal{U}\left({F}_{1}^{{\prime\:}}\right),\mathcal{U}\left({F}_{2}^{{\prime\:}}\right),\mathcal{U}\left({F}_{3}^{{\prime\:}}\right),{F}_{4}^{{\prime\:}}\right)$$

The above process aligns and concatenates representations of different levels in the spatial dimension, enabling the model to simultaneously obtain edge details (from shallow layers) and high-level semantic structures (from deep layers) during the fusion stage.

To avoid information conflicts or redundant interference during the fusion process, the concatenated features $\:{F}_{c}$ are passed through a convolutional module for dimension compression and semantic fusion:

16 $$\:{F}_{m}={\text{C}\text{o}\text{n}\text{v}}_{3\times\:3}\left({F}_{c}\right)$$

This operation not only reduces the channel dimension but also enhances the coupling and integration capability between features, forming a more discriminative fused representation.

### Attention map dynamic balance module (AMDBM)

**Fig. 4:**
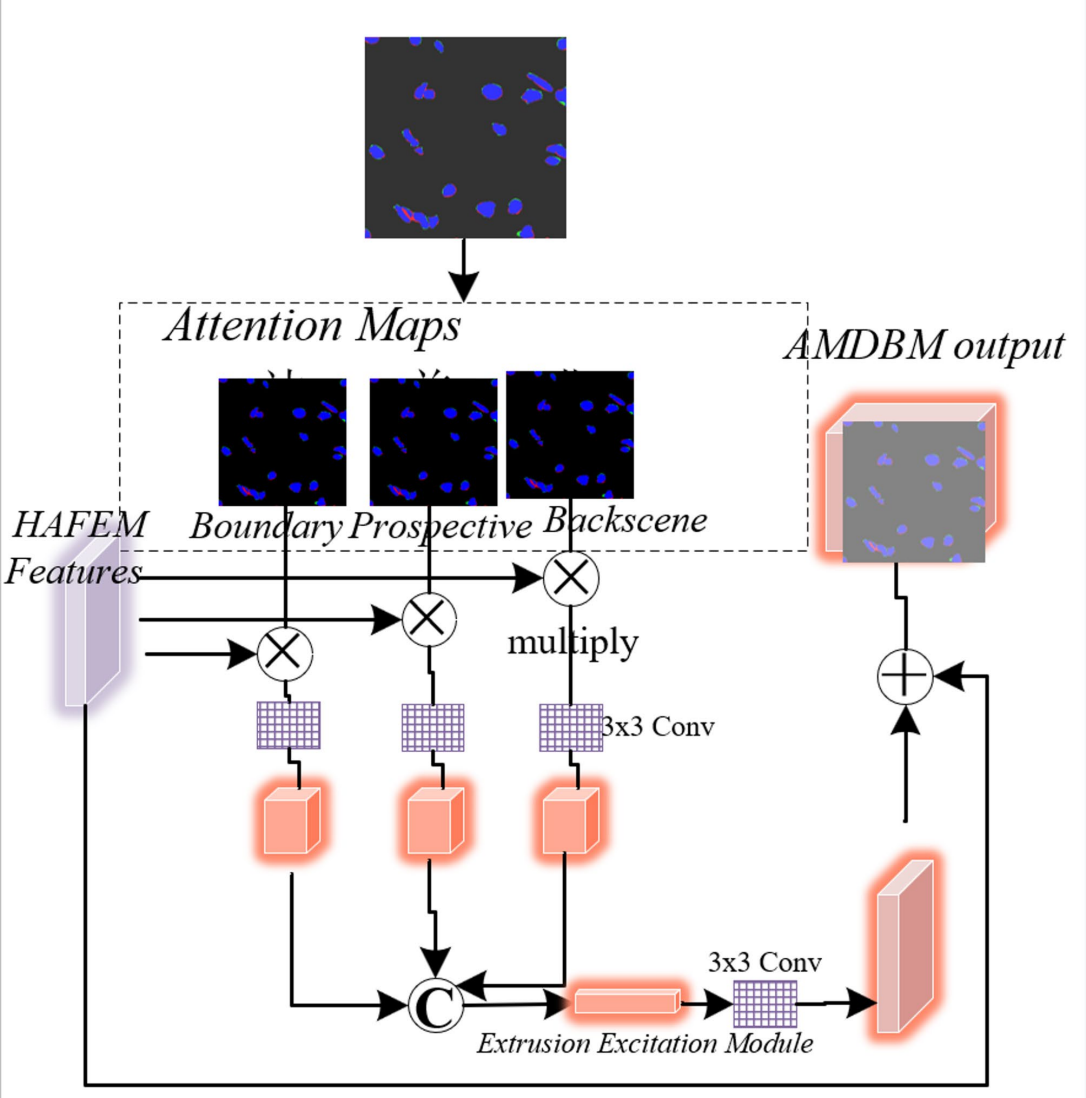
Structure of the AMDBM module.

Digital pathological images often contain interference from fragmented cell nuclei, blank backgrounds, and noise such as shadows and lesions^33–36^. The Attention Map Dynamic Balance Module (AMDBM) calculates attention maps for cell nucleus foregrounds, backgrounds, and boundaries respectively, enabling the network to focus more on the edge details of cell nuclei, eliminating interference from background noise during nucleus segmentation, and making lesion boundaries clearer and smoother. Figure 4 illustrates the AMDBM module’s architecture. The lesion boundary attention score $\:{SC}_{bo}$ ​ generated by the decoder undergoes pixel-wise multiplication with the HAFEM module’s output feature map $\:{F}_{c}$​, producing the boundary-enhanced feature map $\:{F}_{bo}$. This operation is formalized as:

17 $$\:{SC}_{bo}=1-\frac{\left|\sigma\:\left(pred\right)-0.5\right|}{0.5}$$

18 $$\:{F}_{bo}={F}_{c}\otimes\:{SC}_{bo}$$

where $\:\text{p}\text{r}\text{e}\text{d}$ represents the nucleus segmentation prediction result output by the decoder.

The decoder-generated foreground attention score $\:{SC}_{fg}$ is then multiplied pixel-wise with the HAFEM’s output feature map $\:{F}_{c}$​, yielding the foreground-enhanced feature map $\:{F}_{lg}$, formalized as:

19 $$\:{SC}_{fg}=\left[1-\sigma\:\left(pred\right)-{SC}_{bo}\right]$$

20 $$\:{F}_{lg}={F}_{c}\otimes\:{SC}_{fg}$$

Here, $\:{\left[\right]}_{0}^{1}$​ clips attention scores to the [0,1] range. Subsequently, the decoder-derived background attention score $\:{SC}_{bg}$​ undergoes pixel-wise multiplication with the AMDBM module’s output feature map $\:{F}_{c}$, yielding the background-enhanced feature map $\:{F}_{bg}$​, formalized as:

21 $$\:{SC}_{bg}=\left[1-\sigma\:\left(pred\right)-{SC}_{bo}\right]$$

22 $$\:{F}_{bg}={F}_{c}\otimes\:{SC}_{bg}$$

The three feature maps are concatenated and fused, then processed through a squeeze-and-excitation module coupled with 3 × 3 convolutions to produce the balanced attention output $\:{B}_{i}$. The squeeze-and-excitation module reweights feature channels to equilibrate attention across the three regions.

**Fig. 5:**
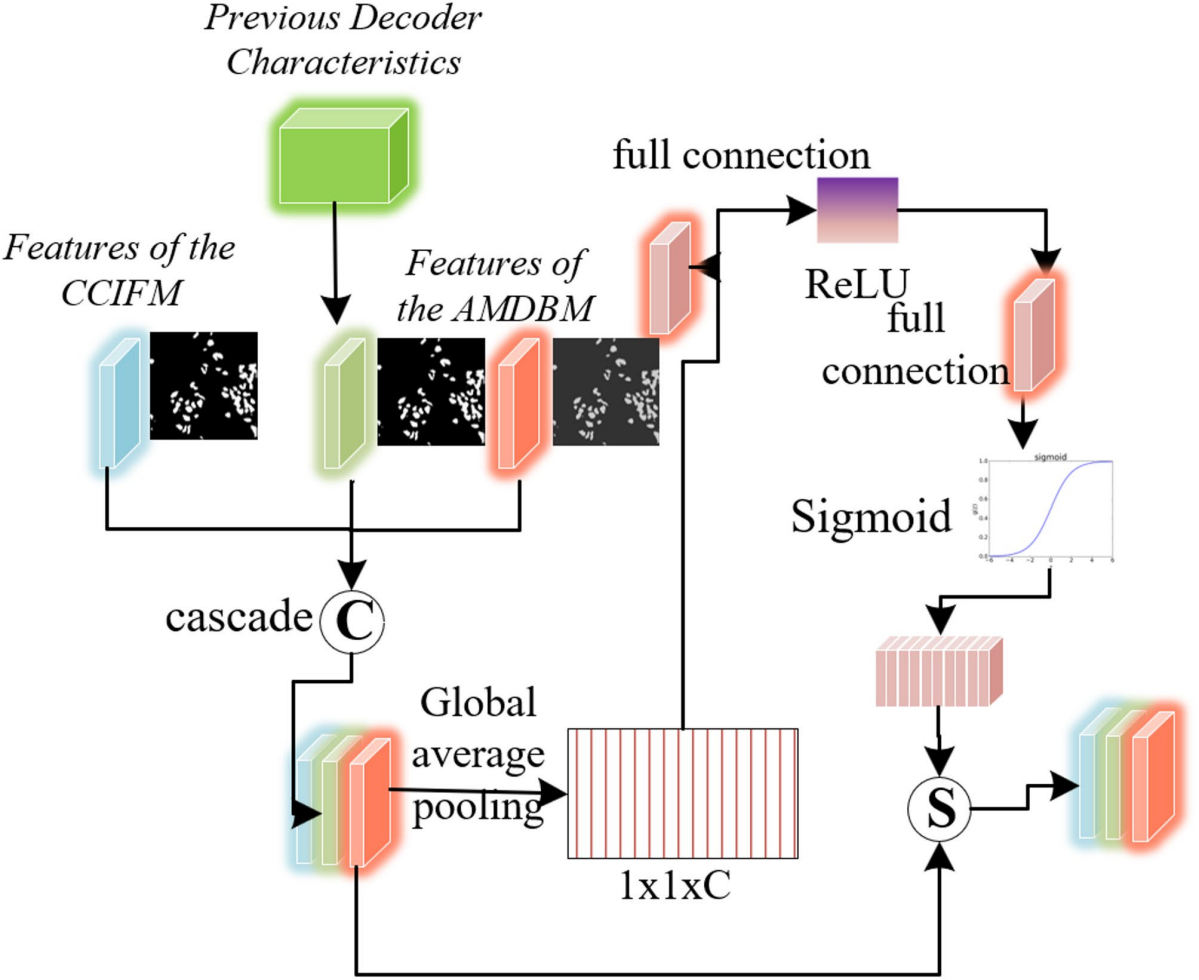
Structure of the AFSM module.

As Fig. 5 depicts, given the significance of local-global context for multi-scale lesions, an Adaptive Feature Selection Module (AFSM) integrates: AMDBM’s attention-balanced features $\:{B}_{i}$, CCIFM’s contextual features $\:{F}_{m}$​, and prior decoder outputs $\:{d}_{i}+1$​. And the AFSM’s resultant features feed into the decoder to enable adaptive contextual feature selection.

The AFSM module is defined as:

23 $$\:{F}_{a}=\text{C}\text{o}\text{n}\text{c}\text{a}\text{t}\left({\text{C}\text{o}\text{n}\text{v}}_{1\times\:1}\left({F}_{m}\right),{\text{C}\text{o}\text{n}\text{v}}_{3\times\:3}^{r=6}\left({F}_{m}\right),{\text{C}\text{o}\text{n}\text{v}}_{3\times\:3}^{r=12}\left({F}_{m}\right),{\text{C}\text{o}\text{n}\text{v}}_{3\times\:3}^{r=18}\left({F}_{m}\right),\text{G}\text{l}\text{o}\text{b}\text{a}\text{l}\text{A}\text{v}\text{g}\text{P}\text{o}\text{o}\text{l}\left({F}_{m}\right)\right)$$

where $\:{Conv}_{3\times\:3}^{r=k}$ represents a dilated convolution with dilation rate *k*. The global average pooling branch is used to introduce image-level context, guiding the network to focus on the overall structure.

Finally, after fusing all scale information, a compression mapping is performed:

24 $$\:{F}_{out}={\text{C}\text{o}\text{n}\text{v}}_{1\times\:1}\left({F}_{a}\right)$$

To obtain the final fused representation $\:{F}_{out}$, which contains rich contextual structure information and multi-scale spatial dependencies, suitable for precise segmentation of complex tumor tissues.

### Loss function

The fused feature $\:{F}_{out}$ is mapped to a probability map through the prediction head.

25 $$\:P=\sigma\:\left({\text{C}\text{o}\text{n}\text{v}}_{1\times\:1}\left({F}_{out}\right)\right)$$

where $\:\sigma\:$ is the Sigmoid activation function, used to output the probability value of each pixel being a lesion. To improve the quality of boundary segmentation, this paper adopts a combined loss function, including Dice loss and BCE (Binary Cross-Entropy) loss.

26 $$\:\text{L}={\uplambda\:}\cdot\:{L}_{\text{D}\text{i}\text{c}\text{e}}+{(1-{\uplambda\:})L}_{\text{B}\text{C}\text{E}}$$

This combined strategy maintains the consistency of the overall region while enhancing the boundary discrimination ability, where $\:\lambda\:\in\:\left[\text{0,1}\right]$ controls the weight of the two.

## Results

### Experimental setup

Osteosarcoma digital pathology image dataset: This study used the digital pathology dataset of osteosarcoma from the Institute of Artificial Intelligence at Monash University^37^, which contains 1000 whole-slice images. Each slice was magnified 40 times, and 512 × 512 sub-images were obtained by random area sampling. After manually removing contaminated or completely blank images, 2164 images were retained for the experiment. Three pathology students then annotated them to ensure the accuracy of the labels. Finally, this study divided the training set, validation set, and test set into a ratio of 7:1:2.

**Fig. 6:**
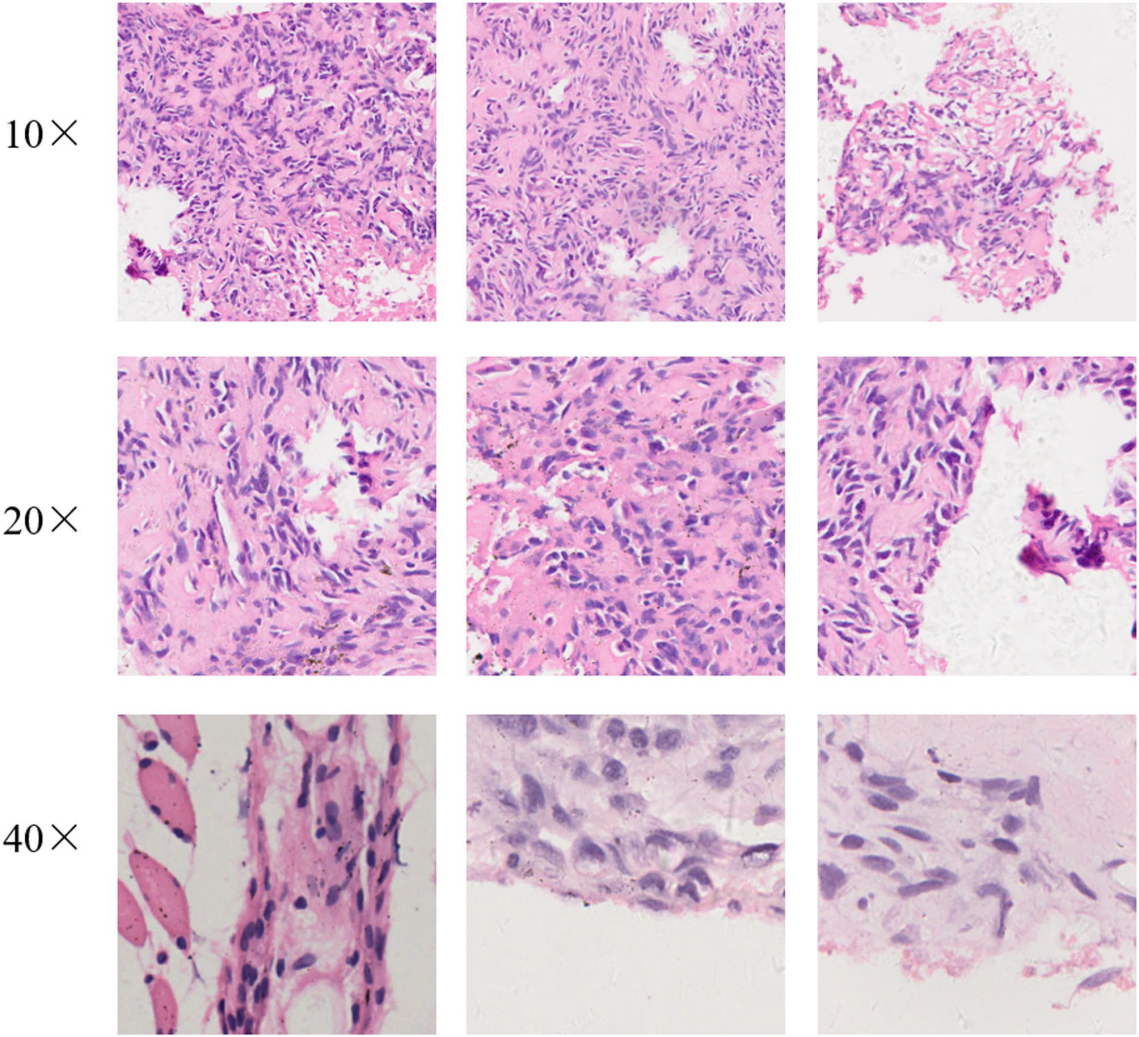
Digital pathology slides at different magnifications.

In addition, in order to verify the performance of the model at lower magnifications, this study magnified the original digital pathology images by 10× and 20× for comparative experiments. Using the same sub-image acquisition method, 1200 10× sub-images and 1200 20× sub-images were obtained respectively. Images of different magnifications are shown in Fig. 6. This study used the same ratio to divide the training set, validation set, and test set.

Public breast cancer dataset TNBC^38^: This data is a collection of digital pathology images selected from triple-negative breast cancer. It contains 33 512 × 512 images.

Experimental Environment: Experiments were conducted on a server with: NVIDIA RTX 3090 GPU, Intel Core i9-12900 K CPU, 64GB RAM, Ubuntu 20.04, and Python 3.8, PyTorch 1.12, CUDA 11.6.

Training process: The Adam optimizer was adopted with an initial learning rate of 1e−4. The training batch size of the model was 32 and a total of 200 training cycles were performed. All input images were normalized before training, while a weighted cross-entropy loss function was chosen to effectively deal with the problem of category imbalance. All experiments were repeated five times and averaged.

Evaluation metrics: In order to provide a more comprehensive and precise quantitative analysis of the segmentation effects of various models, this paper introduces the confusion matrix as an evaluation tool. Based on the above confusion matrix, several key performance indicators can be calculated for evaluating the model performance: accuracy (Acc), precision (Pre), recall (Rec), intersection-union ratio (IoU), Dice Similarity Coefficient (DSC), parameter counts (params), and FLOPs^39^.

### Analysis of results

**Fig. 7:**
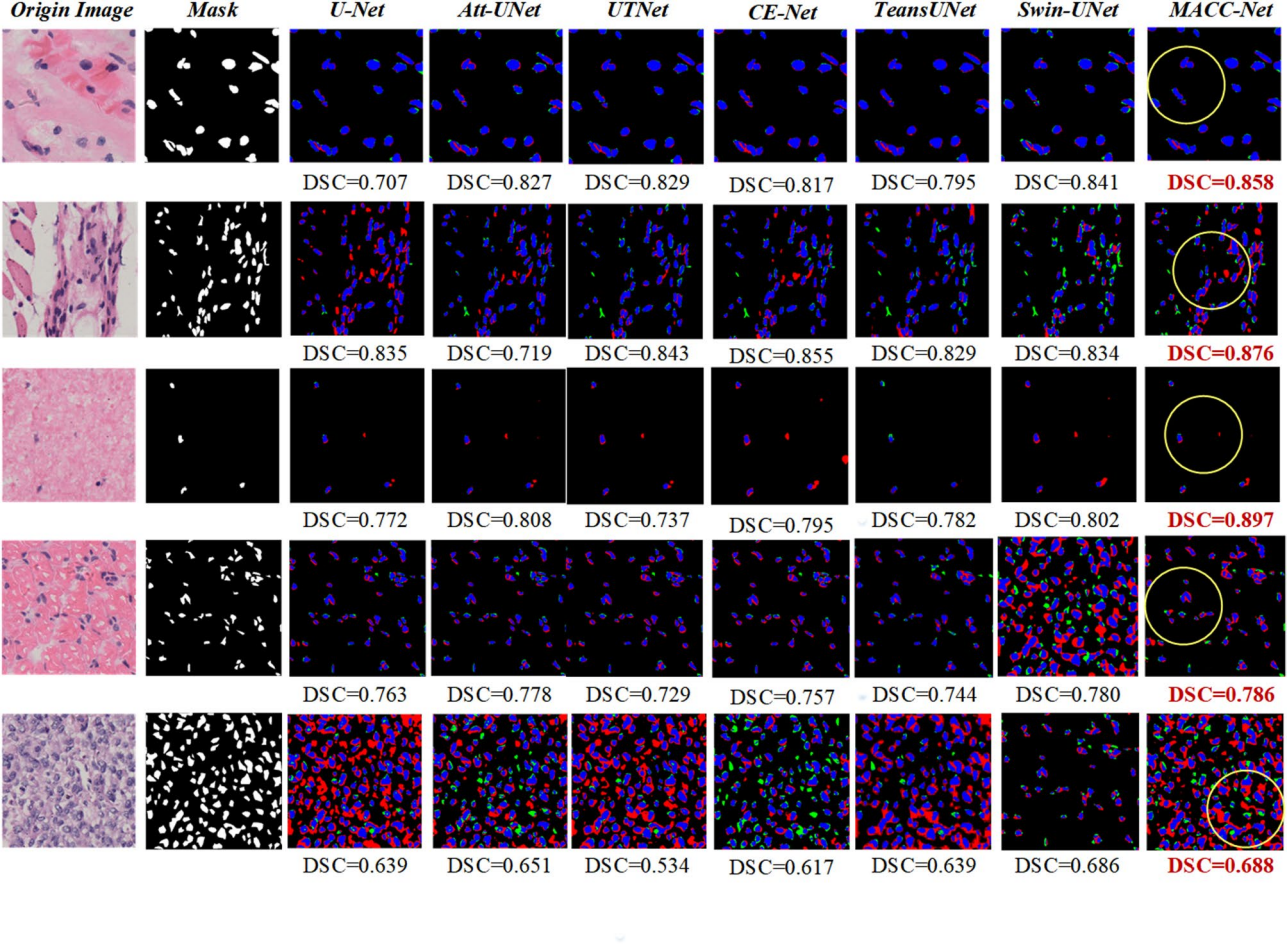
Comparison of the predictive effectiveness of the models. Column 1 displays original pathology images, Column 2 shows ground-truth annotations, and subsequent columns present segmentation outputs from different models.

Figure 7 visually compares osteosarcoma nuclei segmentation results on the test set. Prediction results from multiple sets of images revealed that models like Swin-Unet and TransUnet suffer from missed detections, especially when cell nuclei are densely distributed. The CE-Net model is also more prone to over-segmentation, misclassifying other tissues as cell nuclei, as shown in the third row of figures. Our proposed model, however, excels in cell nucleus recognition, with predictions highly consistent with ground-truth results. It demonstrates robustness and accuracy when dealing with nuclei with complex morphologies or blurred boundaries.

This study quantitatively analyzes the performance of each model in pathological images. The proposed model demonstrating superior segmentation accuracy and higher prediction-label consistency. As detailed in Table 1 (best-performing values bolded), our approach outperforms all counterparts in overall metrics, notably achieving a DSC of 0.847—markedly exceeding other models. The ChannelNet model also performs well, with a DSC value of 0.841. The CE-Net model has the lowest DSC, at only 0.739.

**Table 1:** Comparison of predictive indicators of individual models.

Model	Accuracy	Precision	Recall	F1-score	IOU	DSC
U-Net^41^	0.968	0.881	0.889	0.783	0.651	0.783
Att-Unet^42^	0.976	0.841	0.901	0.825	0.707	0.825
SegNet^43^	0.968	0.898	0.887	0.783	0.652	0.783
UTNet^44^	0.971	0.823	0.835	0.781	0.649	0.781
CE-Net^45^	0.945	0.741	0.832	0.739	0.662	0.739
TransUnet^46^	0.973	0.817	**0.929**	0.809	0.684	0.809
Swin-Unet^47^	0.976	0.856	0.923	0.822	0.702	0.822
ResUnet^48^	0.972	0.878	0.916	0.826	0.709	0.826
ChannelNet^49^	0.979	0.901	0.921	0.841	0.718	0.841
MACC-Net (our)	**0.981**	**0.907**	0.931	**0.847**	0.728	**0.847**

To verify the impact of magnification on model performance, this study selected some models for comparative analysis. As can be seen from Table 2, when the magnification decreases, the image details are significantly reduced, the cell boundaries and microstructures are blurred, the segmentation difficulty increases, and all indicators will decrease. When the digital pathology image is magnified 10 times, the indicators of each model are poor, among which the DSC values of the U-Net and Att-Unet models are lower than 0.7. When the magnification is 20 times, the performance of the model is relatively improved. The DSC value of the ChannelNet model is only 0.801 at 10×; at 20×, it reaches 0.819; at 40×, it reaches 0.841. The DSC value of the MACC-Net model also increased by 4.3% from 0.803. Overall, when the image is magnified 40 times, the performance of the MACC-Net model is the best, and the resource cost of extracting many high-resolution tiles is reasonable. Therefore, the resource cost of extracting many high-resolution images is reasonable.

**Table 2:** Model performance on datasets with different magnifications.

Model	Accuracy				Recall				DSC			
	10×	20×	40×		10×	20×	40×		10×	20×	40×	
U-Net	0.932	0.955	0.968		0.791	0.846	0.889		0.698	0.750	0.783	
Att-Unet	0.955	0.862	0.976		0.826	0.835	0.901		0.661	0.796	0.825	
TransUnet	0.953	0.968	0.973		0.883	0.904	0.929		0.758	0787	0.809	
Swin-Unet	0.956	0.961	0.976		0.876	0.900	0.923		0.782	0.807	0.822	
ChannelNet	0.951	0.966	0.979		0.878	0.899	0.921		0.801	0.819	0.841	
MACC-Net (Our)	0.961	0.975	0.981		0.887	0.908	0.931		0.803	0.826	0.847	

Table 3 shows the nucleus segmentation performance of some models in digital pathology images of different tumors. In the TNBC dataset, the performance of each model was relatively low. The CE-Net model achieved a DSC value of only 0.731. In osteosarcoma, the CE-Net model achieved a DSC value of 0.739. The DSC value of the model in this study also dropped to that of the CE-Net model in the TNBC dataset.

**Table 3:** Model performance in different tumor pathology images.

Model	Accuracy		Recall		DSC	
	40×	TNBC	40×	TNBC	40×	TNBC
SegNet	0.968	0.957	0.887	0.801	0.783	0.761
Swin-Unet	0.976	0.763	0.923	0.856	0.822	0.735
CE-Net	0.945	0.921	0.832	0.876	0.739	0.731
MACC-Net	0.981	0.972	0.931	0.886	**0.847**	0.823

**Fig. 8:**
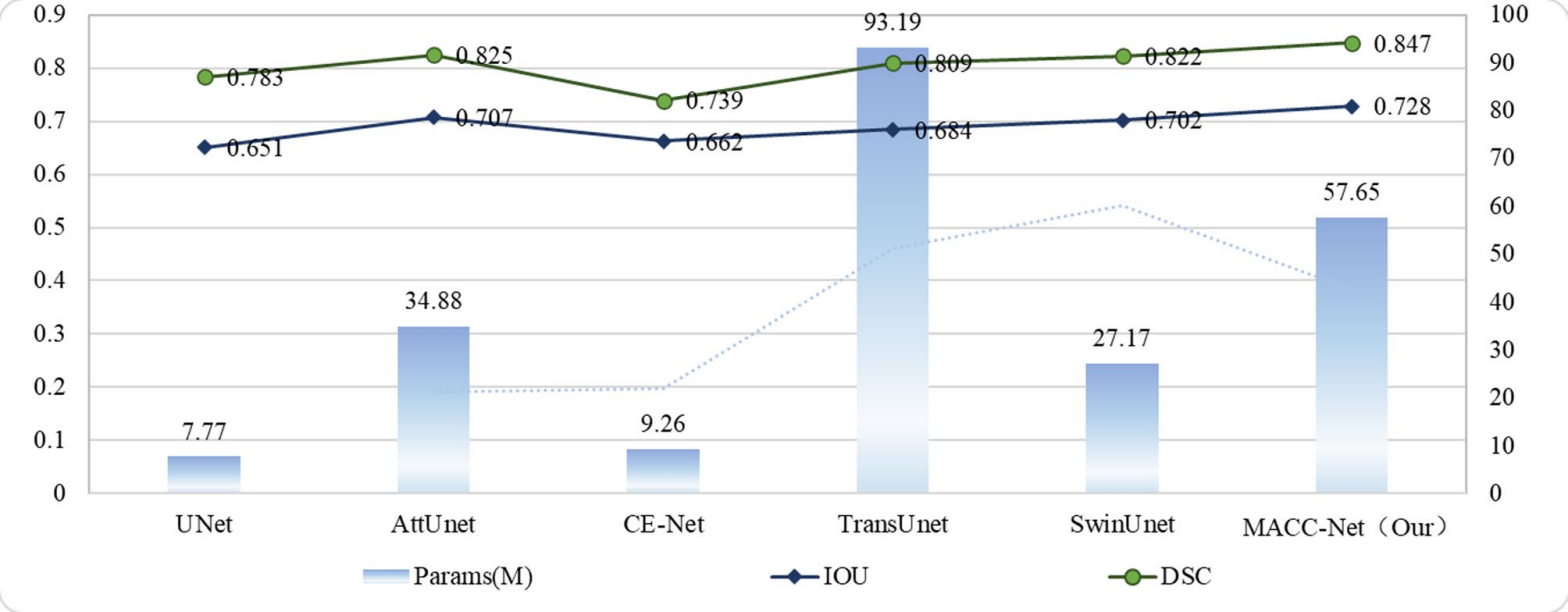
Evaluation of the accuracy and complexity of each model.

**Fig. 9:**
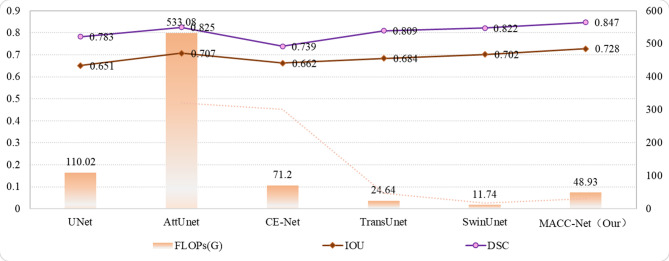
Evaluation of the accuracy and computational complexity of each model.

To more intuitively and comprehensively demonstrate the differences in segmentation performance among different models, this study used pathology images magnified 40× as a benchmark to analyze the relationship between model performance and complexity. Figures 8 and 9 show the relationship between the DSC metric, model parameters, and FLOPs for each segmentation model. As shown in Fig. 8, the MACC-Net model achieves the highest DSC value of all models, reaching 0.847, demonstrating its strong segmentation performance. Furthermore, the MACC-Net model has only 57.65 parameters. Although the UNet and CE-Net models both have well under 10 M parameters, they also perform relatively poorly in terms of segmentation accuracy (DSC and IOU). Furthermore, while the AttUnet model achieves a DSC of 0.825 and an IOU of 0.707, it uses 533 GB of FLOPs, the highest of all models. The SwinUnet model has the lowest FLOPs, at 11.74 GB. The model in this study is only slightly more computationally complex than SwinUnet, but achieves the highest DSC and IOU values. This relatively small increase in computational overhead yields the greatest improvement in accuracy, effectively balancing model accuracy and computational efficiency.

### Ablation experiments

**Fig. 10:**
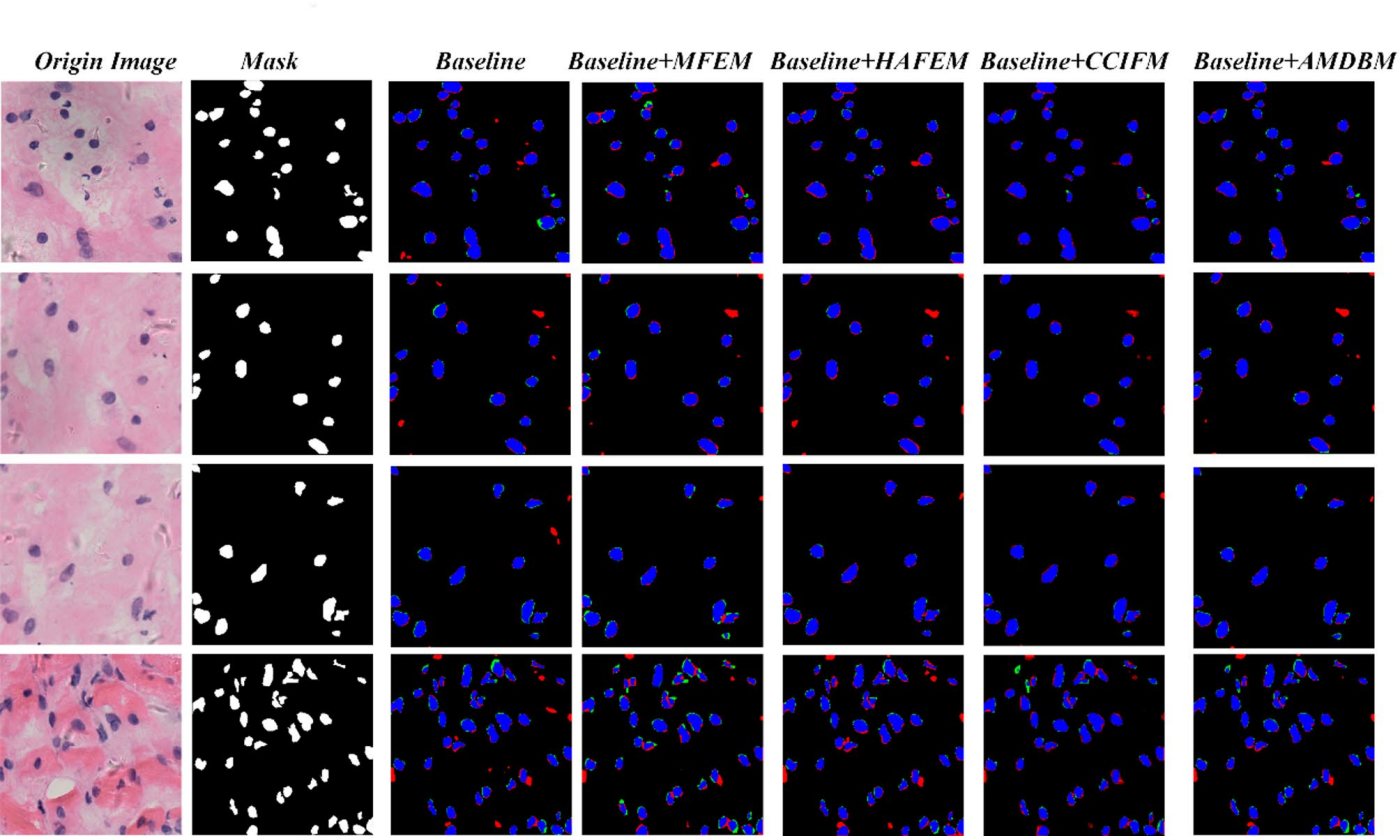
Predictive effect of the model in ablation experiments.

To verify the effectiveness of the modules of the proposed model, we set up eight groups of controlled experiments. Each group of experiments progressively removes or replaces a specific module to observe its effect on the overall performance. The specific experimental settings and results are detailed in Table 4 for the Multiscale Feature Extraction Module (MFEM), the Multiple Attention Enhancement Module (HAFEM), the Cascaded Contextual Fusion Module (CCIFM), and the Balanced Attention Module (AMDBM). From the table, it can be seen that the segmentation performance of the model is improved after adding different modules respectively. The performance improvement of the model is most obvious after combining the three modules. Figure 10 shows the comparison of the prediction effect of the model after adding different modules. The Baseline model has poorer prediction effect and is more prone to misidentification problem. For the case where the background tissues are similar in color to the nuclei, each model may appear to determine the cell tissues as nuclei, as in the picture in the second row. Although the individual models are subject to errors when segmenting densely distributed cell nuclei, the models are relatively the best predictors with the addition of the HAFEM, CCIFM and AMDBM modules.

**Table 4:** Comparison of individual metrics in ablation experiments.

Model	Accuracy	IOU	DSC
Baseline	0.942	0.522	0.586
Baseline + HAFEM	0.957	0.633	0.687
Baseline + CCIFM	0.953	0.623	0.678
Baseline + AMDBM	0.955	0.611	0.677
Baseline + HAFEM + CCIFM	0.964	0.688	0.744
Baseline + HAFEM + AMDBM	0.961	0.656	0.722
Baseline + CCIFM + AMDBM	0.966	0.664	0.731
MACC-Net	**0.981**	0.728	**0.847**

**Fig. 11:**
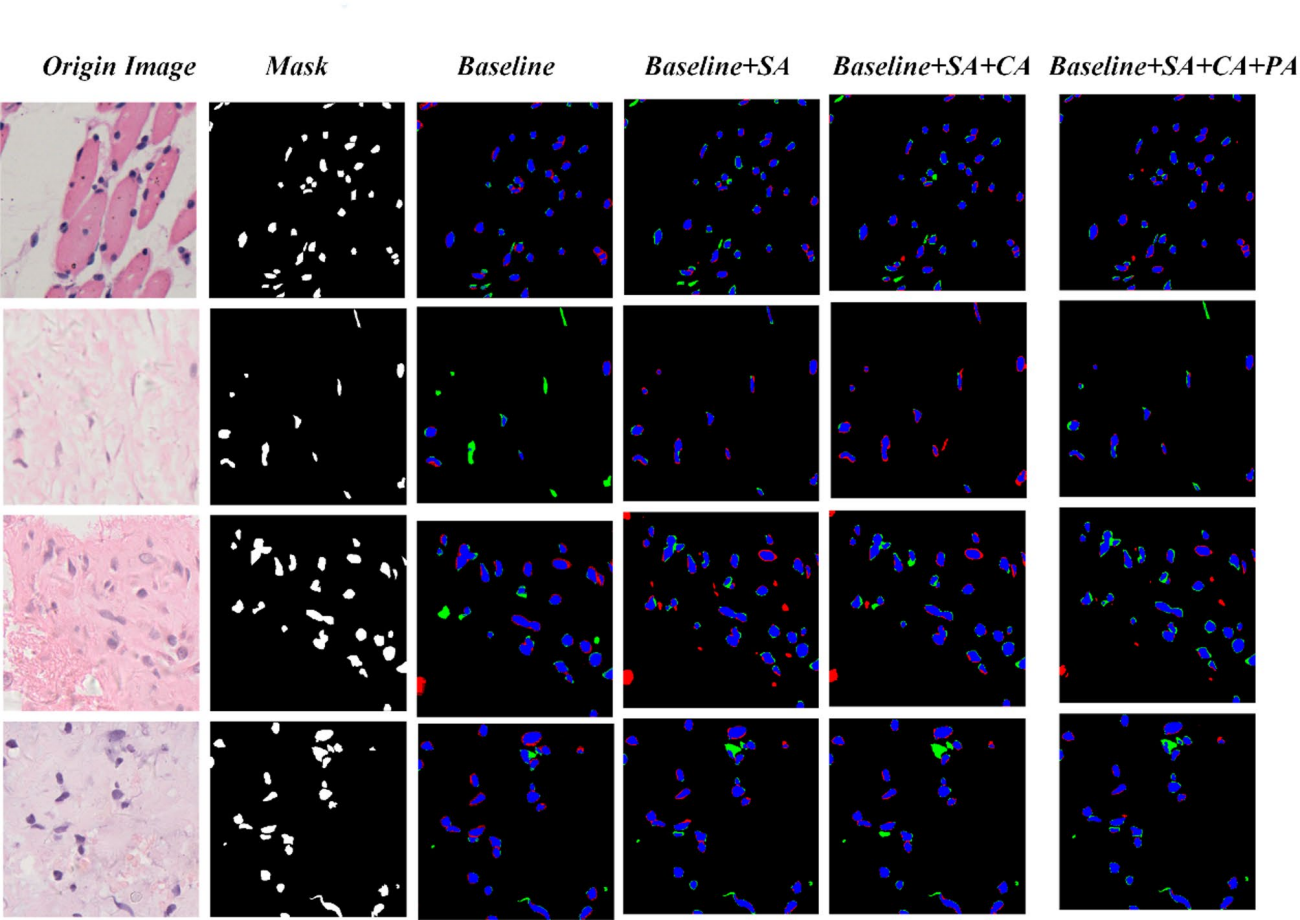
Prediction effect after adding different attention mechanisms.

The performance effects on the model after the addition of different attention mechanisms are shown in Table 5. On the basis of ResNet 50, after adding spatial attention (SA), the model DSC metric improves by 4.6%; after adding only channel attention (CA), the DSC metric suggests 3.7%; after adding channel attention, spatial attention, and pixel-point attention mechanism (PA) at the same time, the IOU metric suggests 7.2% and the DSC metric improves by 1.25. From the outside, we show the prediction effect after adding different attention mechanisms to the model in Fig. 11. The ResNet model is more prone to miss segmentation. Overall, the model’s prediction performance is better after adding channel attention, spatial attention, and pixel attention mechanism.

**Table 5:** Impact of different attention mechanisms on the model.

CA	SA	PA	Accuracy	IOU	DSC
			0.961	0.656	0.722
√			0.965	0.667	0.759
	√		0.966	0.677	0.768
		√	0.957	0.657	0.755
√	√		0.975	0.688	0.812
√		√	0.977	0.694	0.811
	√	√	0.977	0.691	0.807
√	√	√	**0.981**	0.728	**0.847**

## Discussion

The experimental results presented in Chap. 3 (graphs and tables) confirm the superior performance of our approach. As visually evidenced in Fig. 7, the proposed MACC-Net model achieves high accuracy in cell nuclei identification, especially for nuclei with varying scales, complex morphologies, or blurred boundaries. Quantitative comparisons in Table 1 further demonstrate that MACC-Net surpasses other models across multiple evaluation metrics. Notably, it achieves the highest recall, indicating its ability to minimize missed detections while maintaining high sensitivity—a critical advantage for segmentation tasks involving overlapping cells and background interference. This capability enhances its suitability for providing reliable clinical decision support.

Table 2 reveals that segmentation performance is significantly influenced by the magnification levels of digital pathology images. The U-Net model, heavily dependent on local details, exhibits pronounced performance degradation at lower magnifications. In contrast, TransUnet and Swin-Unet, benefiting from global modeling, show greater robustness to resolution variations. Our multi-scale approach maintains relatively stable performance across magnifications. While ChannelNet also performs well, its arbitrary input-to-fixed-three-channel design leads to substantial performance drops when processing low-magnification images with blurred structures.

Computational efficiency and model complexity are critical factors in practical model evaluation. As demonstrated in Figs. 8 and 9, the proposed MACC-Net achieves a favorable balance between accuracy and computational cost, delivering significant performance improvements with relatively low hardware requirements. This makes it particularly suitable for resource-constrained environments. In contrast, while the U-Net model has the lowest computational complexity, its inferior segmentation performance (reflected by its low DSC score) limits its clinical applicability. Other models, such as AttUNet, achieve competitive segmentation accuracy but at the expense of high computational complexity, leading to substantial training resource consumption and elevated deployment costs—factors that hinder their practical adoption in real-world clinical scenarios.

Currently, due to the importance, complexity, and large size of pathology images, whole slide imaging (WSI) plays a crucial role in streamlining pathology workflows, ensuring reproducibility, and dissemination^49–52^. Mark D. Zarella et al. [a–54] provide a detailed review of the applications of WSI in pathology practice, medical education, and research, as well as the challenges and prospects of its implementation. Paola Chiara Rizzo et al.^54^ summarize the applications of pathology in routine clinical diagnosis, categorizing them as “diagnostic” and “non-diagnostic.” Stefano Marletta et al.^55^ review and analyze the literature on placental digital pathology. The widespread application of AI in WSI has significantly promoted reproducibility^56–58^. For example, in the analysis of biological specimen data, AI can integrate diverse data, such as digital images and patient health records, to enhance the comprehensiveness of research or diagnosis^59^. At the same time, Oliwia Koteluk et al.^60^ also raise the potential threat of AI medical tools to medicine. Therefore, AI tools should enhance rather than replace doctors.

## Conclusion and outlook

This study proposes a digital pathology image assisted recognition method (MACC-Net) based on a multi-attention mechanism. This method improves the recognition accuracy of cell nucleus features through multi-dimensional feature extraction and fusion. The results show that this method has better performance in pathology image segmentation and can provide efficient and reliable assistance for clinical pathology diagnosis.

Although this study has a high accuracy rate for cropped standard pathology slices, it is usually necessary to observe complete pathology images in actual clinical environments. This method has problems with splicing artifacts and global information loss. With the improvement of algorithm capabilities and memory, studying full slice processing solutions will be our focus.

## Data Availability

The datasets generated and/or analysed during the current study are not publicly available due to the confidentiality of the data supporting the findings of this study, the results are currently being commercialized, but are available from the corresponding author on reasonable request. The corresponding author will consider data requests 12 months after publication of this article.
